# *N*-(5-Sulfanyl­idene-4,5-dihydro-1,3,4-thia­diazol-2-yl)acetamide dimethyl sulfoxide disolvate

**DOI:** 10.1107/S1600536811054298

**Published:** 2011-12-23

**Authors:** Sung Kwon Kang, Nam Sook Cho, Siyoung Jang

**Affiliations:** a Department of Chemistry, Chungnam National University, Daejeon 305-764, Republic of Korea

## Abstract

In the title compound, C_4_H_5_N_3_OS_2_·2C_2_H_6_OS, the five-membered heterocyclic ring and the N—(C=O)—C plane of the acetamide group are essentially co-planar, with a dihedral angle of 1.25 (3)°. Inter­molecular N—H⋯O hydrogen bonds between the acetamide compound and the dimethyl sulfoxide mol­ecules stabilize the crystal structure. The two dimethyl sulfoxide mol­ecules are each disordered over two positions with occupancy ratios of 0.605 (2):0.395 (2) and 0.8629 (18):0.1371 (18).

## Related literature

For the synthesis and biological activity of thia­diazole compounds, see: Hildebrandt *et al.* (2011 ▶); Cho *et al.* (1993 ▶). For the structures of thia­diazole derivatives, see: Zhan *et al.* (2007 ▶, 2009 ▶).
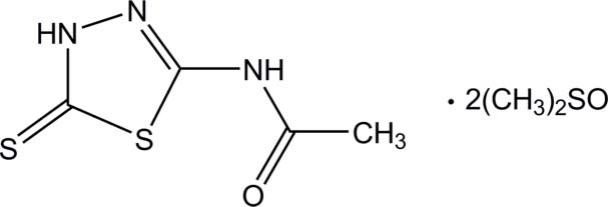


## Experimental

### 

#### Crystal data

C_4_H_5_N_3_OS_2_·2C_2_H_6_OS
*M* *_r_* = 331.49
Triclinic, 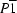

*a* = 7.090 (2) Å
*b* = 9.982 (3) Å
*c* = 11.513 (3) Å
α = 100.872 (6)°
β = 96.827 (4)°
γ = 91.359 (4)°
*V* = 793.6 (4) Å^3^
*Z* = 2
Mo *K*α radiation
μ = 0.60 mm^−1^
*T* = 296 K
0.28 × 0.18 × 0.13 mm

#### Data collection

Bruker SMART CCD area-detector diffractometer
Absorption correction: multi-scan (*SADABS*; Bruker, 2002 ▶) *T*_min_ = 0.894, *T*_max_ = 0.916
24389 measured reflections
3292 independent reflections
2625 reflections with *I* > 2σ(*I*)
*R*_int_ = 0.180

#### Refinement

*R*[*F* ^2^ > 2σ(*F* ^2^)] = 0.039
*wR*(*F* ^2^) = 0.117
*S* = 1.06
3292 reflections
245 parameters
7 restraints
H atoms treated by a mixture of independent and constrained refinement
Δρ_max_ = 0.22 e Å^−3^
Δρ_min_ = −0.35 e Å^−3^

### 

Data collection: *SMART* (Bruker, 2002 ▶); cell refinement: *SAINT* (Bruker, 2002 ▶); data reduction: *SAINT*; program(s) used to solve structure: *SHELXS97* (Sheldrick, 2008 ▶); program(s) used to refine structure: *SHELXL97* (Sheldrick, 2008 ▶); molecular graphics: *ORTEP-3* (Farrugia, 1997 ▶); software used to prepare material for publication: *WinGX* (Farrugia, 1999 ▶).

## Supplementary Material

Crystal structure: contains datablock(s) global, I. DOI: 10.1107/S1600536811054298/is5026sup1.cif

Structure factors: contains datablock(s) I. DOI: 10.1107/S1600536811054298/is5026Isup2.hkl

Supplementary material file. DOI: 10.1107/S1600536811054298/is5026Isup3.cml

Additional supplementary materials:  crystallographic information; 3D view; checkCIF report

## Figures and Tables

**Table 1:** Hydrogen-bond geometry (Å, °)

*D*—H⋯*A*	*D*—H	H⋯*A*	*D*⋯*A*	*D*—H⋯*A*
N5—H5⋯O16	0.88 (2)	1.91 (2)	2.783 (3)	170 (3)
N7—H7⋯O12	0.86 (2)	1.89 (2)	2.734 (8)	166 (2)

**Fig. 1.**
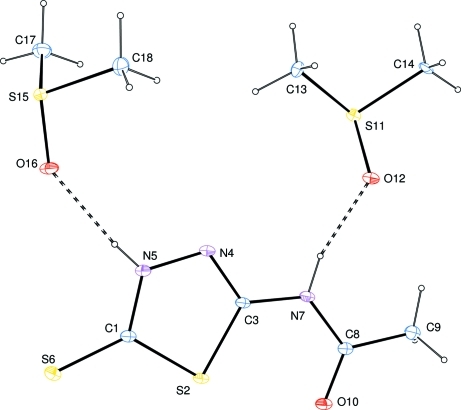
Molecular structure of the title compound, showing the atom-numbering scheme and 30% probability ellipsoids. DMSO molecules show only major parts. Intermolecular N—H···O hydrogen bonds are indicated by dashed lines.

**Fig. 2.**
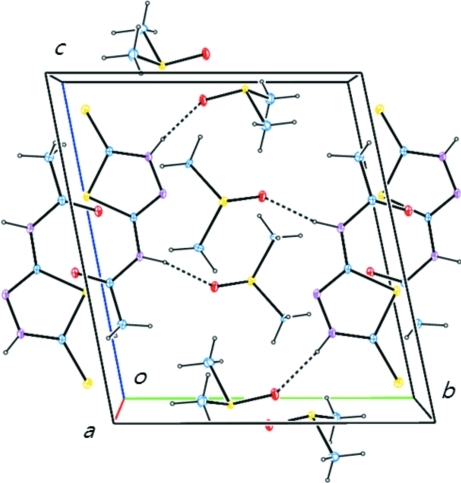
Part of the crystal structure of the title compound, showing molecules linked by intermolecular N—H···O hydrogen bonds (dashed lines).

## supplementary crystallographic information

### Comment

Thiadiazole derivatives have recently attracted attention in synthesis and biological activities (Hildebrandt *et al.*, 2011; Zhan *et al.*, 2009; Zhan *et al.*, 2007). Our interest in thiadiazoles have formed systematic efforts to obtain new biologically active pyrimidines, purines and their analogs (Cho *et al.*, 1993). 5-Amino-2*H*-1,2,4-thiadiazol-3-one is five-membered ring analog of cytosine. 5-Amino-3*H*-1,3,4-thiadiazole-2-thione is a sulfur analog of 5-amino-3*H*-1,3,4-thiadizol-2-one which is an isomer of 5-amino-2*H*-1,2,4-thiadiazol-3-one. Herein, the crystal structure of acetylation of 5-amino-3*H*-1,3,4-thiadiazole-2-thione, (I), is reported (Fig. 1).

The 1,3,4-thiadiazol-2-yl five-membered ring is planar, with a mean deviation of 0.008 Å from the corresponding least-squares plane defined by the seven constituent atoms. The bond distance of C3—N4 [1.2952 (23) Å] is shorter than that of N4—C1 [1.3365 (22) Å], which is consistent with double bond character. The dihedral angle between the 5-thioxo-1,3,4-thiadiazol-2-yl heterocyclic ring and the acetamide group is 1.25 (3) °, which is essentially planar. The crystal structure is stabilized by the intermolecular N—H···O hydrogen bonds between the compound and the DMSO molecules (Fig. 2 and Table 1).

### Experimental

5-Amino-3*H*-1,3,4-thiadiazole-2-thione (1.33 g, 0.011 mol) was dissolved in tetrahydrofuran (50 ml). Triethylamine(1.51 g, 0.015 mol) and a methyl benzoyl chloride (0.01 mol) were added to the solution and the mixture was refluxed with stirring for 4 h. Triethylamine hydrochloride was filtered off, the solution was concentrated to one-third of its original volume, and carefully acidified with concentrated hydrochloric acid. The precipitate was collected by filteration and recrystallized from aqueous ethanol to obtain an analytical product. Colorless crystals of (I) were obtained from its DMSO solution by slow evaporation of the solvent at room temperature.

### Refinement

Atoms H5 and H7 of the NH groups were located in a difference Fourier map and refined freely. Other H atoms were positioned geometrically and refined using a riding model, with C—H = 0.96 Å, and with *U*_iso_(H) = 1.5*U*_eq_(carrier C) for methyl H atoms. Two DMSO molecules are disordered with occupancy ratio, 0.605 (2):0.395 (2) and 0.8629 (18):0.1371 (18). Distance restraints [C—S = 1.81 (2) Å and S═O = 1.50 (2) Å] were applied for the DMSO molecules in the refinement.

### Crystal data

**Table:**

C_4_H_5_N_3_OS_2_·2C_2_H_6_OS	*Z* = 2
*M**_r_* = 331.49	*F*(000) = 348
Triclinic, *P*1	*D*_x_ = 1.387 Mg m^−^^3^
Hall symbol: -P 1	Mo *K*α radiation, λ = 0.71073 Å
*a* = 7.090 (2) Å	Cell parameters from 8583 reflections
*b* = 9.982 (3) Å	θ = 2.5–27.9°
*c* = 11.513 (3) Å	µ = 0.60 mm^−^^1^
α = 100.872 (6)°	*T* = 296 K
β = 96.827 (4)°	Block, colourless
γ = 91.359 (4)°	0.28 × 0.18 × 0.13 mm
*V* = 793.6 (4) Å^3^	

### Data collection

**Table:**

Bruker SMART CCD area-detector diffractometer	2625 reflections with *I* > 2σ(*I*)
graphite	*R*_int_ = 0.180
φ and ω scans	θ_max_ = 26.5°, θ_min_ = 1.8°
Absorption correction: multi-scan (*SADABS*; Bruker, 2002)	*h* = −8→8
*T*_min_ = 0.894, *T*_max_ = 0.916	*k* = −12→12
24389 measured reflections	*l* = −14→14
3292 independent reflections	

### Refinement

**Table:**

Refinement on *F*^2^	Primary atom site location: structure-invariant direct methods
Least-squares matrix: full	Secondary atom site location: difference Fourier map
*R*[*F*^2^ > 2σ(*F*^2^)] = 0.039	Hydrogen site location: inferred from neighbouring sites
*wR*(*F*^2^) = 0.117	H atoms treated by a mixture of independent and constrained refinement
*S* = 1.06	*w* = 1/[σ^2^(*F*_o_^2^) + (0.0538*P*)^2^ + 0.016*P*] where *P* = (*F*_o_^2^ + 2*F*_c_^2^)/3
3292 reflections	(Δ/σ)_max_ < 0.001
245 parameters	Δρ_max_ = 0.22 e Å^−^^3^
7 restraints	Δρ_min_ = −0.35 e Å^−^^3^

### Special details

**Table:**

Geometry. All s.u.'s (except the s.u. in the dihedral angle between two l.s. planes) are estimated using the full covariance matrix. The cell s.u.'s are taken into account individually in the estimation of s.u.'s in distances, angles and torsion angles; correlations between s.u.'s in cell parameters are only used when they are defined by crystal symmetry. An approximate (isotropic) treatment of cell s.u.'s is used for estimating s.u.'s involving l.s. planes.
Refinement. Refinement of *F*^2^ against ALL reflections. The weighted *R*-factor *wR* and goodness of fit *S* are based on *F*^2^, conventional *R*-factors *R* are based on *F*, with *F* set to zero for negative *F*^2^. The threshold expression of *F*^2^ > 2σ(*F*^2^) is used only for calculating *R*-factors(gt) *etc*. and is not relevant to the choice of reflections for refinement. *R*-factors based on *F*^2^ are statistically about twice as large as those based on *F*, and *R*- factors based on ALL data will be even larger.

### Fractional atomic coordinates and isotropic or equivalent isotropic displacement parameters (Å^2^)

**Table:**

	*x*	*y*	*z*	*U*_iso_*/*U*_eq_	Occ. (<1)
C1	0.2794 (2)	0.12725 (17)	0.77693 (18)	0.0551 (4)	
S2	0.26666 (7)	0.02071 (4)	0.63771 (4)	0.05621 (17)	
C3	0.2555 (2)	0.16373 (16)	0.57197 (17)	0.0504 (4)	
N4	0.2592 (2)	0.27936 (14)	0.64572 (16)	0.0618 (4)	
N5	0.2732 (2)	0.25591 (15)	0.75980 (16)	0.0604 (4)	
H5	0.277 (4)	0.323 (2)	0.822 (2)	0.113 (10)*	
S6	0.29892 (10)	0.07416 (6)	0.90641 (5)	0.0767 (2)	
N7	0.2429 (2)	0.15853 (15)	0.45173 (15)	0.0566 (4)	
H7	0.238 (3)	0.237 (2)	0.432 (2)	0.070 (6)*	
C8	0.2374 (3)	0.03883 (18)	0.36972 (18)	0.0577 (5)	
C9	0.2250 (3)	0.0526 (2)	0.2426 (2)	0.0721 (6)	
H9A	0.2405	0.1472	0.2382	0.108*	
H9B	0.103	0.0168	0.2017	0.108*	
H9C	0.3234	0.0026	0.2058	0.108*	
O10	0.2408 (2)	−0.07095 (13)	0.40190 (14)	0.0768 (4)	
S11	0.14717 (14)	0.51941 (8)	0.37275 (8)	0.0627 (4)	0.605 (2)
O12	0.2642 (8)	0.3906 (8)	0.3592 (8)	0.0824 (17)	0.605 (2)
C13	0.265 (2)	0.6272 (13)	0.5029 (11)	0.101 (4)	0.605 (2)
H13A	0.2399	0.5925	0.5721	0.151*	0.605 (2)
H13B	0.2205	0.7179	0.5081	0.151*	0.605 (2)
H13C	0.4	0.6296	0.4989	0.151*	0.605 (2)
C14	0.2160 (13)	0.6060 (7)	0.2625 (7)	0.068 (3)	0.605 (2)
H14A	0.1725	0.5532	0.1848	0.102*	0.605 (2)
H14B	0.352	0.6181	0.2719	0.102*	0.605 (2)
H14C	0.1607	0.6936	0.271	0.102*	0.605 (2)
S11A	0.3122 (2)	0.52142 (13)	0.37732 (12)	0.0645 (5)	0.395 (2)
O12A	0.1799 (11)	0.3913 (11)	0.3575 (12)	0.076 (2)	0.395 (2)
C13A	0.231 (3)	0.6329 (17)	0.4958 (14)	0.080 (4)	0.395 (2)
H13D	0.2722	0.6043	0.5693	0.12*	0.395 (2)
H13E	0.0943	0.6316	0.4838	0.12*	0.395 (2)
H13F	0.2811	0.7239	0.499	0.12*	0.395 (2)
C14A	0.220 (3)	0.6115 (17)	0.2720 (14)	0.114 (7)	0.395 (2)
H14D	0.2269	0.5592	0.1938	0.171*	0.395 (2)
H14E	0.2912	0.6967	0.2821	0.171*	0.395 (2)
H14F	0.0893	0.6289	0.2815	0.171*	0.395 (2)
S15	0.30211 (9)	0.63050 (5)	0.96900 (5)	0.0570 (2)	0.8629 (18)
O16	0.2521 (8)	0.4816 (3)	0.9404 (3)	0.0775 (9)	0.8629 (18)
C17	0.0886 (7)	0.7112 (4)	0.9385 (3)	0.0873 (8)	0.8629 (18)
H17A	0.0047	0.6992	0.9957	0.131*	0.8629 (18)
H17B	0.029	0.6716	0.8598	0.131*	0.8629 (18)
H17C	0.1156	0.807	0.9433	0.131*	0.8629 (18)
C18	0.4168 (7)	0.6718 (6)	0.8504 (5)	0.096 (2)	0.8629 (18)
H18A	0.5394	0.6329	0.8514	0.144*	0.8629 (18)
H18B	0.432	0.7692	0.8598	0.144*	0.8629 (18)
H18C	0.341	0.6355	0.7758	0.144*	0.8629 (18)
S15A	0.2077 (7)	0.6133 (4)	0.8695 (4)	0.0785 (16)	0.1371 (18)
O16A	0.235 (6)	0.4742 (18)	0.8952 (17)	0.085 (7)	0.1371 (18)
C17A	0.136 (4)	0.735 (3)	0.9939 (13)	0.084 (9)	0.1371 (18)
H17D	0.0093	0.7106	1.0059	0.126*	0.1371 (18)
H17E	0.141	0.8248	0.9765	0.126*	0.1371 (18)
H17F	0.2215	0.732	1.0647	0.126*	0.1371 (18)
C18A	0.444 (3)	0.679 (2)	0.876 (3)	0.074 (9)	0.1371 (18)
H18D	0.5276	0.6042	0.8642	0.111*	0.1371 (18)
H18E	0.4821	0.7358	0.9524	0.111*	0.1371 (18)
H18F	0.4509	0.7311	0.8146	0.111*	0.1371 (18)

### Atomic displacement parameters (Å^2^)

**Table:**

	*U*^11^	*U*^22^	*U*^33^	*U*^12^	*U*^13^	*U*^23^
C1	0.0548 (10)	0.0387 (8)	0.0736 (12)	0.0039 (7)	0.0097 (8)	0.0141 (8)
S2	0.0670 (3)	0.0319 (2)	0.0721 (3)	0.00558 (19)	0.0085 (2)	0.0157 (2)
C3	0.0503 (9)	0.0337 (8)	0.0705 (12)	0.0047 (7)	0.0102 (8)	0.0162 (7)
N4	0.0803 (11)	0.0341 (7)	0.0735 (11)	0.0070 (7)	0.0120 (8)	0.0148 (7)
N5	0.0752 (10)	0.0376 (8)	0.0700 (11)	0.0056 (7)	0.0123 (8)	0.0125 (7)
S6	0.1065 (5)	0.0548 (3)	0.0725 (4)	0.0054 (3)	0.0090 (3)	0.0227 (3)
N7	0.0644 (9)	0.0363 (7)	0.0729 (11)	0.0053 (6)	0.0091 (7)	0.0196 (7)
C8	0.0561 (10)	0.0457 (9)	0.0719 (12)	0.0033 (8)	0.0072 (9)	0.0132 (8)
C9	0.0815 (14)	0.0619 (12)	0.0734 (14)	0.0056 (10)	0.0078 (11)	0.0155 (10)
O10	0.1132 (12)	0.0372 (7)	0.0806 (10)	0.0058 (7)	0.0118 (9)	0.0129 (6)
S11	0.0750 (8)	0.0494 (5)	0.0673 (6)	−0.0013 (4)	0.0127 (4)	0.0184 (4)
O12	0.120 (4)	0.0437 (17)	0.093 (3)	0.019 (3)	0.030 (4)	0.0259 (16)
C13	0.151 (8)	0.073 (6)	0.066 (5)	0.012 (4)	−0.019 (4)	0.000 (4)
C14	0.116 (6)	0.040 (3)	0.057 (4)	−0.003 (3)	0.007 (3)	0.032 (3)
S11A	0.0768 (12)	0.0559 (8)	0.0666 (8)	0.0172 (6)	0.0152 (6)	0.0216 (6)
O12A	0.115 (6)	0.036 (2)	0.079 (4)	0.008 (4)	0.003 (5)	0.020 (2)
C13A	0.123 (9)	0.061 (7)	0.064 (8)	0.008 (5)	0.031 (7)	0.020 (5)
C14A	0.151 (16)	0.097 (9)	0.089 (10)	0.037 (8)	0.025 (9)	−0.004 (7)
S15	0.0831 (4)	0.0407 (3)	0.0477 (4)	0.0060 (2)	0.0121 (3)	0.0067 (2)
O16	0.117 (2)	0.0372 (13)	0.080 (2)	0.0071 (13)	0.026 (2)	0.0065 (14)
C17	0.096 (2)	0.0585 (18)	0.11	0.0158 (16)	0.019 (3)	0.019 (2)
C18	0.126 (4)	0.093 (3)	0.073 (3)	−0.013 (3)	0.045 (3)	0.012 (2)
S15A	0.106 (3)	0.056 (2)	0.068 (3)	0.000 (2)	−0.002 (2)	0.0071 (18)
O16A	0.159 (16)	0.023 (6)	0.064 (13)	−0.004 (7)	0.029 (14)	−0.021 (7)
C17A	0.15 (3)	0.077 (14)	0.039 (8)	0.048 (15)	0.054 (13)	0.010 (10)
C18A	0.103 (16)	0.034 (9)	0.067 (15)	0.016 (9)	−0.030 (12)	−0.013 (8)

### Geometric parameters (Å, °)

**Table:**

C1—N5	1.337 (2)	S11A—C13A	1.754 (15)
C1—S6	1.666 (2)	C13A—H13D	0.96
C1—S2	1.740 (2)	C13A—H13E	0.96
S2—C3	1.7367 (17)	C13A—H13F	0.96
C3—N4	1.295 (2)	C14A—H14D	0.96
C3—N7	1.368 (2)	C14A—H14E	0.96
N4—N5	1.370 (2)	C14A—H14F	0.96
N5—H5	0.878 (17)	S15—O16	1.486 (4)
N7—C8	1.373 (2)	S15—C17	1.762 (4)
N7—H7	0.86 (2)	S15—C18	1.777 (5)
C8—O10	1.221 (2)	C17—H17A	0.96
C8—C9	1.488 (3)	C17—H17B	0.96
C9—H9A	0.96	C17—H17C	0.96
C9—H9B	0.96	C18—H18A	0.96
C9—H9C	0.96	C18—H18B	0.96
S11—O12	1.540 (7)	C18—H18C	0.96
S11—C14	1.772 (5)	S15A—O16A	1.485 (18)
S11—C13	1.777 (12)	S15A—C18A	1.772 (19)
C13—H13A	0.96	S15A—C17A	1.822 (14)
C13—H13B	0.96	C17A—H17D	0.96
C13—H13C	0.96	C17A—H17E	0.96
C14—H14A	0.96	C17A—H17F	0.96
C14—H14B	0.96	C18A—H18D	0.96
C14—H14C	0.96	C18A—H18E	0.96
S11A—O12A	1.548 (11)	C18A—H18F	0.96
S11A—C14A	1.720 (12)		
			
N5—C1—S6	127.50 (16)	S11A—C13A—H13E	109.5
N5—C1—S2	107.67 (15)	H13D—C13A—H13E	109.5
S6—C1—S2	124.82 (10)	S11A—C13A—H13F	109.5
C3—S2—C1	89.24 (9)	H13D—C13A—H13F	109.5
N4—C3—N7	121.00 (16)	H13E—C13A—H13F	109.5
N4—C3—S2	115.01 (15)	S11A—C14A—H14D	109.5
N7—C3—S2	124.00 (13)	S11A—C14A—H14E	109.5
C3—N4—N5	109.17 (14)	H14D—C14A—H14E	109.5
C1—N5—N4	118.91 (17)	S11A—C14A—H14F	109.5
C1—N5—H5	119 (2)	H14D—C14A—H14F	109.5
N4—N5—H5	121.9 (19)	H14E—C14A—H14F	109.5
C3—N7—C8	123.36 (16)	O16—S15—C17	105.9 (3)
C3—N7—H7	113.9 (15)	O16—S15—C18	107.7 (3)
C8—N7—H7	122.7 (15)	C17—S15—C18	97.3 (3)
O10—C8—N7	120.52 (19)	S15—C17—H17A	109.5
O10—C8—C9	123.44 (18)	S15—C17—H17B	109.5
N7—C8—C9	116.04 (17)	H17A—C17—H17B	109.5
C8—C9—H9A	109.5	S15—C17—H17C	109.5
C8—C9—H9B	109.5	H17A—C17—H17C	109.5
H9A—C9—H9B	109.5	H17B—C17—H17C	109.5
C8—C9—H9C	109.5	S15—C18—H18A	109.5
H9A—C9—H9C	109.5	S15—C18—H18B	109.5
H9B—C9—H9C	109.5	H18A—C18—H18B	109.5
O12—S11—C14	104.0 (4)	S15—C18—H18C	109.5
O12—S11—C13	103.8 (6)	H18A—C18—H18C	109.5
C14—S11—C13	99.9 (5)	H18B—C18—H18C	109.5
S11—C13—H13A	109.5	O16A—S15A—C18A	102.8 (19)
S11—C13—H13B	109.5	O16A—S15A—C17A	113.6 (12)
H13A—C13—H13B	109.5	C18A—S15A—C17A	98.3 (14)
S11—C13—H13C	109.5	S15A—C17A—H17D	109.5
H13A—C13—H13C	109.5	S15A—C17A—H17E	109.5
H13B—C13—H13C	109.5	H17D—C17A—H17E	109.5
S11—C14—H14A	109.5	S15A—C17A—H17F	109.5
S11—C14—H14B	109.5	H17D—C17A—H17F	109.5
H14A—C14—H14B	109.5	H17E—C17A—H17F	109.5
S11—C14—H14C	109.5	S15A—C18A—H18D	109.5
H14A—C14—H14C	109.5	S15A—C18A—H18E	109.5
H14B—C14—H14C	109.5	H18D—C18A—H18E	109.5
O12A—S11A—C14A	104.9 (8)	S15A—C18A—H18F	109.5
O12A—S11A—C13A	104.7 (7)	H18D—C18A—H18F	109.5
C14A—S11A—C13A	93.7 (9)	H18E—C18A—H18F	109.5
S11A—C13A—H13D	109.5		

### Hydrogen-bond geometry (Å, °)

**Table:**

*D*—H···*A*	*D*—H	H···*A*	*D*···*A*	*D*—H···*A*
N5—H5···O16	0.88 (2)	1.91 (2)	2.783 (3)	170 (3)
N7—H7···O12	0.86 (2)	1.89 (2)	2.734 (8)	166 (2)

## References

[bb1] Bruker (2002). *SADABS*, *SAINT* and *SMART* Bruker AXS Inc., Madison, Wisconsin, USA.

[bb2] Cho, N. S., Kim, G. N. & Parkanyi, C. (1993). *J. Heterocycl. Chem.* **30**, 397–401.

[bb3] Farrugia, L. J. (1997). *J. Appl. Cryst.* **30**, 565.

[bb4] Farrugia, L. J. (1999). *J. Appl. Cryst.* **32**, 837–838.

[bb5] Hildebrandt, A., Schaarschmidt, D., van As, L., Swarts, J. C. & Lang, H. (2011). *Inorg. Chim. Acta*, **374**, 112–118.

[bb6] Sheldrick, G. M. (2008). *Acta Cryst.* A**64**, 112–122. 10.1107/S0108767307043930 18156677

[bb7] Zhan, P., Liu, X., Fang, Z., Li, Z., Pannecouque, C. & De Clercq, E. (2009). *Eur. J. Med. Chem.* **44**, 4648–4653. 10.1016/j.ejmech.2009.06.037 19628308

[bb8] Zhan, J.-Y., Xiong, D.-J., Wang, Y.-G. & Li, H.-B. (2007). *Acta Cryst.* E**63**, o2184–o2185.

